# The Chamber for Studying Rice Response to Elevated Nighttime Temperature in Field

**DOI:** 10.1155/2013/647897

**Published:** 2013-09-05

**Authors:** Song Chen, Xi Zheng, Dangying Wang, Chunmei Xu, Ma. Rebecca C. Laza, Xiufu Zhang

**Affiliations:** ^1^China National Rice Research Institute, Chinese Academy of Agricultural Sciences, Hangzhou, Zhejiang 310006, China; ^2^985-Institute of Agrobiology and Environmental Sciences, Zhejiang University, Hangzhou 310029, China; ^3^Crop, Soil, and Water Sciences Division, International Rice Research Institute, P.O. Box 7777, Metro Manila, Philippines

## Abstract

An *in situ* temperature-controlled field chamber was developed for studying a large population of rice plant under different nighttime temperature treatments while maintaining conditions similar to those in the field during daytime. The system consists of a pipe hoop shed-type chamber with manually removable covers manipulated to provide a natural environment at daytime and a relatively stable and accurate temperature at night. Average air temperatures of 22.4 ± 0.3°C at setting of 22°C, 27.6 ± 0.4°C at 27°C, and 23.8 ± 0.7°C ambient conditions were maintained with the system. No significant horizontal and vertical differences in temperature were found and only slight changes in water temperatures were observed between the chambers and ambient conditions at 36 days after transplanting. A slight variation in CO_2_ concentration was observed at the end of the treatment during the day, but the 10-**μ**mol CO_2_ mol^−1^ difference was too small to alter plant response. The present utilitarian system, which only utilizes an air conditioner/heater, is suitable for studying the effect of nighttime temperature on plant physiological responses with minimal perturbation of other environmental factors. At the same time, it will enable *in situ* screening of many rice genotypes.

## 1. Introduction

Global warming can affect the function of crops in the natural environment. Increased nighttime temperatures can impact the growth and development of crops, and they have been implicated in the decrease in crop yield in tropical and other regions [1]. Nighttime temperature has been predicted to rise more than daytime temperature in the near future [2]. Most studies on the effect of temperature on rice growth were done under temperature-controlled chambers using pot-grown plants with a limited number of genotypes. This is because growing of crops in the field on a large scale under controlled temperature requires a precise apparatus to control night temperature. A temperature-controlled field chamber (TCFC) is ideal for studying a large population of rice plants under different night air temperature treatments while maintaining conditions similar to those in field in the daytime. In addition, a large crop population is desirable to avoid the physiological and statistical deviations obtained from using a smaller population. Plant physiological experimentation involves both environmental variables and ambient conditions plus or minus some proportion or magnitude of the environmental factor, depending on the objectives of the study [3]. To facilitate studies on plant physiological responses to high nighttime temperature, accurate temperature control is essential. Responses of plant populations are multiple, and a well-replicated experiment is necessary to clearly distinguish these responses. Thus, the apparatus or system to be used should avoid unintended effects on the local environment.

Current facilities used to study the effects of high night temperatures are limited to controlling the elevated temperature on the small plant population and with a few or no replications. Besides, plants are exposed to simulated artificial environmental conditions such as light, wind, and relative humidity. Greenhouses, growth chambers, phytotrons, open-top chambers (OTC), infrared heater (IR) systems, and soil-plant-atmosphere-research (SPAR) units are usually used in controlled environmental studies [3]. Each of these temperature-controlled facilities, which can be used on small plant populations and pot-grown plants, has certain limitations. Greenhouses generally have higher humidity, lower wind speed, and lower light intensity than natural conditions outside. Moreover, greenhouse coverings typically transmit two-thirds to three-fourths of available sunlight [4]. Temperature in artificially lighted growth chambers is well controlled; however, plants are subjected to an environment with artificial light. The phytotron has light conditions similar to those of artificially lighted growth chambers, but it has smaller rooting volumes, which might restrict the partitioning of carbohydrates to the roots [5]. Open-top chambers require a high air flow rate in and out of the chamber to control temperature and humidity [4]. However, some studies have shown that daytime and nighttime temperatures in OTCs were higher compared with the neighboring unenclosed areas [6, 7]. The IR system has the advantage in terms of accuracy and precision of temperature control including reliability in system performance and mobility, but its limitation has something to do with thermal radiation efficiency and cost in warming larger field crop populations [8, 9]. Finally, the SPAR units are one of the best in controlling environmental factors [3], but the cost and the limited portability of the units make them site-specific. Most of the above mentioned facilities can elevate or control nighttime temperatures, but they fail to adjust to changes in light, humidity, and other factors, resulting in poor simulation of natural environmental conditions [10]. Modified growth chambers that can precisely control nighttime temperature without altering daytime natural environmental conditions such as light intensity, humidity, and wind speed are ideal for conducting studies on the effect of elevated temperature on rice plants *in situ*.

As mentioned, growth chambers or greenhouses, particularly those with large temperature differences, have apparent differences in sunlight intensity, wind speed, humidity, and CO_2_, which can confound the effect of temperature on the plant physiology. We developed new chambers particularly designed for nighttime temperature research with minimal effect on other environmental factors but the temperature at night. Here, we describe in detail the design and construction of the modified chamber and present profiles of system performance.

## 2. Materials and Method

### 2.1. Chamber Design

**Figure 1:**
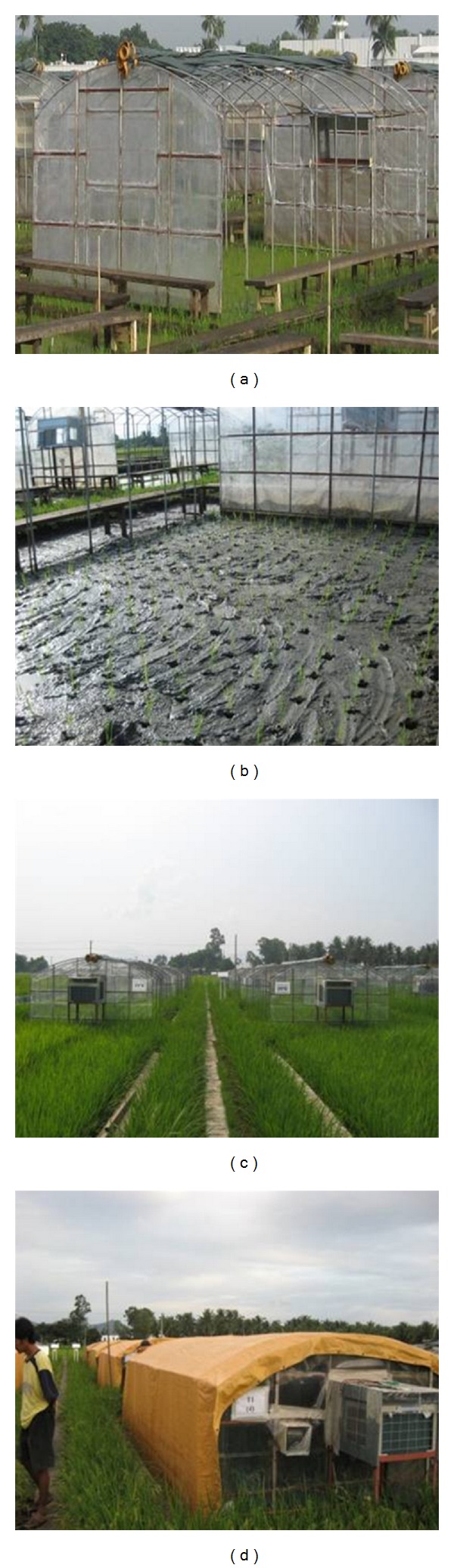
The actual setup of a field chamber located at the IRRI farm. (a) Single chamber; (b) inside of chamber with rice seedling; (c) profile of the chamber systems in field. (d) Chamber covered by canvas after 6PM.

The system was installed at the International Rice Research Institute (IRRI) farm in Los Baños, Laguna (121.15 E, 14.1 N, and 10 m above the sea level), Philippines. Sixteen field chambers oriented to the north-south direction were constructed at the experimental site. The pipe hoop shed-type structure similar in design to a commercial greenhouse was adopted (Figure 1). Chamber dimensions were 5.6 m (length), 3.0 m (width), 1.5 m (height), 0.5 m (arch height), and 30° (arc). The framework consisted of a series of shed-type pipe. The front and back were of aluminum steel pipes structured as a skeleton, covered with a layer of insulation of UV-transparent film (0.05 mm thick ethylene-tetrafluoroethylene copolymer (ETFE) film, WAH PHIL corporation), which had a transparency of 65–87% wavelengths between 250 and 700 nm. The films were fixed on the frameworks with metal fittings. During the daytime, the shading of the frame was minimal because the open sides of the chamber are oriented to the east. Moreover, two rows of rice plants around the edge were set as the border (boundary row) to avoid the possible error caused by the system. Thirty to fourty centimeters pipes were drilled into the soil along the side of the chamber to support the shed-type pipes. Pipes with an outer diameter of 20.0 mm were bolted at 50 cm intervals. The pipe structure was reinforced with screw or welding. An air conditioner/heater (CW-1803VPH, Matsushita Electric Philippines Corporation) was placed at 90 cm above the ground level outside of chamber. This was supported by an aluminum sheet to protect the equipment from too much sun exposure and to reduce heat loss. Two inlet fans (HEWF-8, Myhanabishi Corporation) were installed 90 cm above the ground in the front (north) frame, and two outlet fans were installed at 135 cm at the back (south) frame. The distance between the two fans was 102 cm. Each chamber was spaced at 2.8 m interval to ensure proper ventilation and avoid mutual shading. Eight plots under ambient conditions served as control.

### 2.2. Control and Maintenance of Desired Temperature

During the night temperature treatment, the field chambers were covered with insulating plastic canvas attached to a long cylindrical wood and the chamber perimeter was enclosed by pulling the side canvas downward below the water level (2-3 cm deep) to prevent ambient air from entering the chamber along its entire lengths. After treatment, the canvas was rolled up horizontally into a line and placed at the midtop of the chamber. The closing and opening of canvas cover were done manually. The times of chamber closing and opening are set to typical sunset and sunrise times during the treatment periods. The cover was closed at 6:00 PM and opened at 6:00 AM during the treatment periods.

The temperature gradient in each chamber was generated by an air-conditioning/heating system equipped with a controller (CW-1805V, Matsushita Electric Philippines Corp., Taytay, Rizal, Philippines). To maintain the desired high nighttime temperature, hot air was generated from the heater and circulated by inlet/outlet fans. These fans not only facilitated the distribution of hot or cold air but also allowed air exchange inside and outside the chamber. The fan was selected in terms of wind speed (about 1 m/s at 1 m distance from the fan), depending on the room space and the desired temperature. The various increases in chamber temperature were simulated to match the natural seasonal variation of ambient air temperature. Differences in water vapor and CO_2_ concentration between chamber and ambient conditions were minimized by the air exchange through the exhaust fans.

### 2.3. System Performance Check

System performance was evaluated in a field experiment conducted at the IRRI experimental farm in 2009 dry season (DS). Two rice genotypes of IR72 and IR8 were used. Crop management followed the standard cultural practices. Fourteen-day-old seedlings, raised on seedling trays, were manually transplanted on 9 January in the DS at a hill spacing of 0.2 m × 0.2 m with four seedlings per hill. All fertilizers were manually broadcasted, and incorporation was made only for basal application. 30 kg P ha^−1^, 40 kg K ha^−1^, and 5 kg Zn ha^−1^ were applied as basal fertilizers. Nitrogen in the form of urea was split-applied 60 kg ha^−1^ at basal, 40 kg ha^−1^ at midtillering, 60 kg ha^−1^ at panicle initiation, and 40 kg ha^−1^ at flowering. Nighttime temperatures inside the chambers were set at 22°C (T22), 27°C (T27), and ambient. During daytime, the chambers were not covered and were thus exposed to natural environmental conditions.

Grain yields were obtained from a central 5 m^2^ harvest area in each sampling plot at harvestable maturity. Grain sub-samples from the harvested area were oven-dried to constant weight at 70°C. 12 plants were obtained from the harvest area and manually threshed into rachis and spikelets. Filled spikelets and unfilled spikelets were separated by submerging in tap water then oven-dried at 70°C to constant weight to determine dry weight. Grain filling rate was calculated as the ratio of the filled grain to the total grains.

#### 2.3.1. Air and Water Temperatures and Relative Humidity

The interaction of humidity and temperature affects rice yield because of their influence on spikelet fertility. Air temperature, RH, and vapor pressure deficit (VPD) differences between the T22 and T27 and between those and ambient conditions during the cropping season were measured using T/RH sensors (HOBO Onset computer Corp., Bourne, MA, USA) installed in each chamber. The measurements started at 36 days after transplanting (DAT) from 7 PM to 6 AM. For water temperature, measurement was also taken at 3-4 DAT (without a standing crop). An additional sensor (placed at a 3 cm water depth) was used to determine water temperature. Values of VPD were calculated according to Prenger and Ling's formula (2001) [11]:

(1) $$\begin{array}{c} \text{V}{\text{P}}_{\mathrm{sat}}=e^{(A/T)+B+CT+{DT}^{2}+{ET}^{3}+F\ln T}\;\text{KPa}. \end{array}$$

Here, *A* = −1.88 × 10^4^, *B* = −13.1, *C* = −1.5 × 10^−2^, *D* = 8 × 10^−7^, *E* = −1.69 × 10^−11^, and *F* = 6.456; and *T* is the temperature of the air in *K*, *T* (*K*) = *T* (°C) + 273.15.

So,

(2) $$\begin{array}{c} \text{VPD}\;\left(\%\right)=\text{V}{\text{P}}_{\mathrm{sat}}\times \frac{\text{relative}\;\;\text{humidity}}{100}. \end{array}$$

#### 2.3.2. Temperature Distribution inside the Chamber

A three-dimensional array of sensors were placed in the chamber to examine the positional differences in temperature. Both horizontal and vertical temperature/RH profiles were measured by distributing six sensors along the north-south axis (1.5 m intervals) at different heights (0.5 m and 1.5 m).

#### 2.3.3. CO_2_ Concentration

Carbon dioxide inside the T22 and T27 chambers and ambient conditions were measured 3-4 days before transplanting (without a standing crop) and at 36 DAT using the Li-Cor-6400 (Li-Cor, Lincoln, NE, USA) portable gas exchange system. Measurements of CO_2_ were done at 10 PM and 5 AM. Sensors were also installed to monitor water temperature (at 3 cm water depth).

### 2.4. Data Record and Analysis

All temperature and RH data sets in the chamber were monitored individually every 10 s and averaged over 5 min intervals by the HOBO data logger/sensor. The controller/data logger system was operated by the HOBOware pro. These data sets can be manipulated simply, even if one has no knowledge of technical programming. Data were analyzed by using Microsoft excel 2003 and SAS 8.0 (2003). Means and standard deviations/standard errors are reported for each of the measurements.

## 3. Result and Discussion

### 3.1. Temperature Gradient between the Chambers

**Figure 2:**
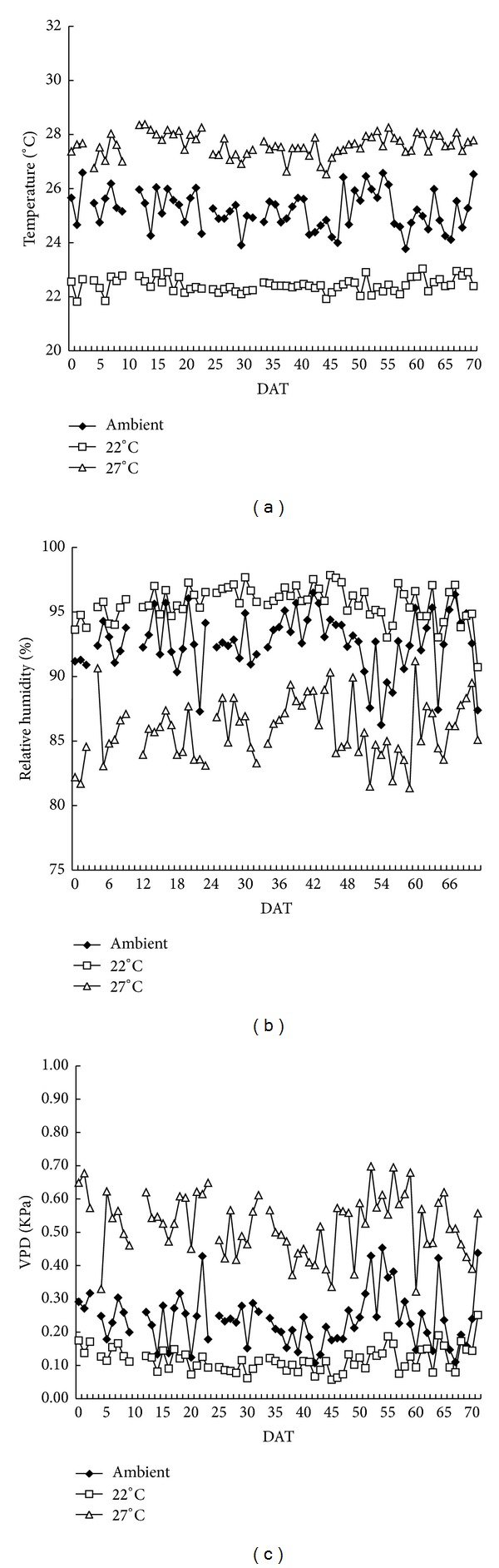
Dynamic changes in daily average air temperature (a) relative humidity, (b) and VPD (c) of the days after transplanting (DFT) in 2009 DS. The data was obtained from the sensors at 1.5 m above water level in treatments of 22°C (□), 27°C (△), and ambient (◆).

**Figure 3:**
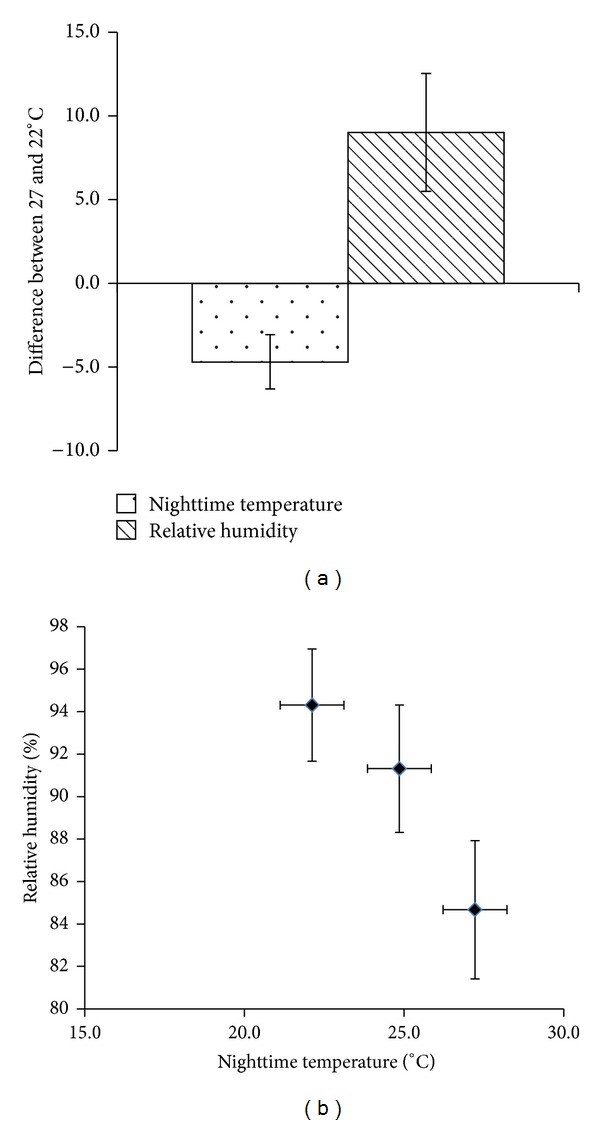
Difference in nighttime temperature and relative humidity between 22°C and 27°C in 2009 DS. The data was obtained from the sensors at 1.5 m above water level.

A satisfactory air temperature in the chamber was achieved and maintained throughout the 2009 experiment (Figure 2(a)). Average nighttime air temperatures of 22.4 ± 0.3°C (T22), 27.6 ± 0.4°C (T27), and the 23.8 ± 0.7°C (ambient) were recorded. A temperature gap of 5°C between the low- and high-temperature treatments was realized with a precision of ±0.4°C. However, the lowest and highest daily means air temperatures in T22, T27, and ambient had relatively large variations during the whole season. The lowest to highest temperature range were 19.6°C to 23.0°C in T22, 26.5°C to 28.4°C in T27, and 21.8°C to 26.6°C in ambient. The reliability of air temperature and humidity were shown in Figure 3.

**Figure 4:**
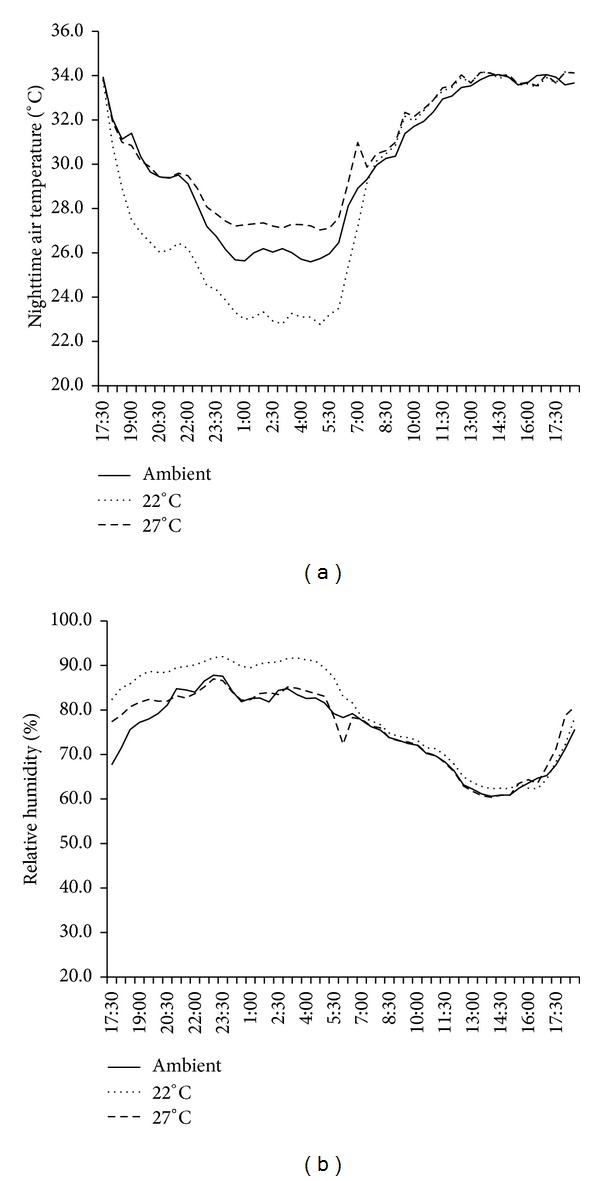
Typical diurnal patterns in air temperature and relative humidity for ambient conditions, 22°C, and 27°C in 2009 DS. The data was obtained from the sensors at 1.5 m above water level.

An acceptable humidity in the chamber was also maintained throughout the season (Figure 2(b)). The nighttime RH during the cropping season was 95.7 ± 1.3% in T22, 85.8 ± 2.4% in T27, and 92.6 ± 2.3% in ambient conditions. Variations in RH between the low and high temperatures were minimized at 9.2 ± 3.4% in this system. Relative humidity differed between the simulated and ambient environments exists; however, we believed that this difference will not influence plant growth because the negative effect of high RH (more than 80%) on spikelet fertility under high temperature, owing to the interruption of pollination process, occurs during daytime [12]. Our treatments were carried out at nighttime, but pollination commonly occurred between 9 am and 12 pm. Thus, RH influence was avoided by the experimental design. Moreover, in the chamber, the mean RH in T27 (85.8%) was smaller than that in ambient conditions (92.6%) and T22 (95.7%). The difference was within the 10% range, which was too small to influence the effect of temperature on plant growth as shown by the slight decline in fertility percentage during daytime with RH more than 75% [12]. One of the most important concerns in using a controlled-temperature environment is the dynamics of atmospheric vapor pressure. During the cropping season, VPD was 0.52 ± 0.09 KPa in T27, 0.12 ± 0.04 KPa in T22, and 0.24 ± 0.08 KPa in ambient conditions (Figure 2(c)). The incoming air was mixed with the released heated or cooled air inside the chamber. A lower VPD in T22 and a higher VPD in T27 compared with the ambient VPD was observed. The mean VPD in T22 was significantly lower than that in T27, which may be attributed to elevated temperature and consequently may result in the differential physiological response of the plants. Typical diurnal patterns in air temperature and relative humidity for ambient conditions, 22°C and 27°C were shown in Figure 4.

### 3.2. Positional Differences in Temperature and Relative Humidity in the Chamber

**Figure 5:**
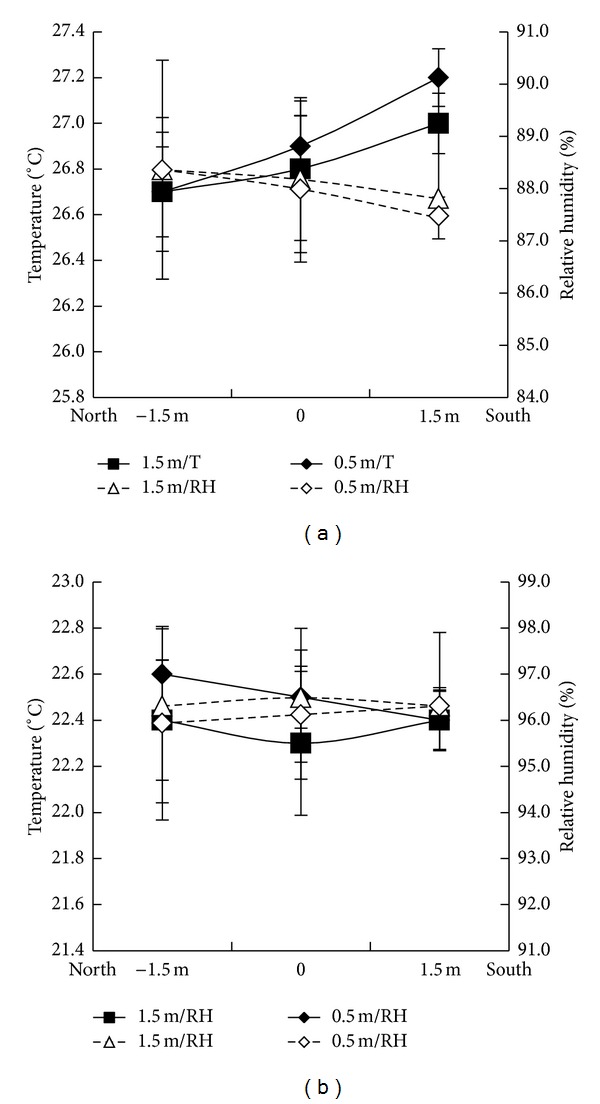
Temperature and relative humidity profiles across the horizontal axis (1.5 m, 3.0 m, and 4.5 m distances from air conditioner and heater (north to south direction)) at 0.5 m and 1.5 m above ground level) in T27 (a) and T22 (b) chambers 3 days before transplanting. Error bars are SE (*n* = 4 replicates).

Three-dimensional tests were carried out in the chambers T27 and T22. Differences in RH and temperature between 0.5 m and 1.5 m height above the water level were not significant in both T27 and T22 chambers. The average temperatures in chamber T27 were 26.7°C, 26.8°C, and 27.1°C, and the RH values were 88.4%, 88.1%, and 87.5% along the heated airflow. However, the difference was not statistically significant. Similarly, in chamber T22, the average temperature/RH was 22.4°C/96.5%, 22.5°C/96.7%, and 22.4°C/96%, along the cooled airflow. No significant differences in temperature and RH were found in both directions across chambers (Figure 5).

### 3.3. Chamber and Ambient Water Temperatures

Water temperature is also an important consideration in using the field chamber system as the setup is established in the rice field where paddy water is in its natural state. The water temperatures of T27, T22, and ambient conditions were examined before transplanting (without plants) and at 36 DAT (with plants) to determine the effect of air temperature on water temperature inside the chambers (Table 1). Water temperature in T27 (28.6 ± 2.1°C) was significantly higher than that of T22 (27.1 ± 0.57°C), whereas the differences between ambient conditions and T22 were not significant. Before transplanting (without plants), the elevated air temperature in T27 significantly increased water temperature compared with ambient conditions; however, the difference was no longer significant at 36 DAT (Table 1).

### 3.4. CO_2_ Concentrations at Different Chamber Temperatures before Transplanting and 36 DAT

**Figure 6:**
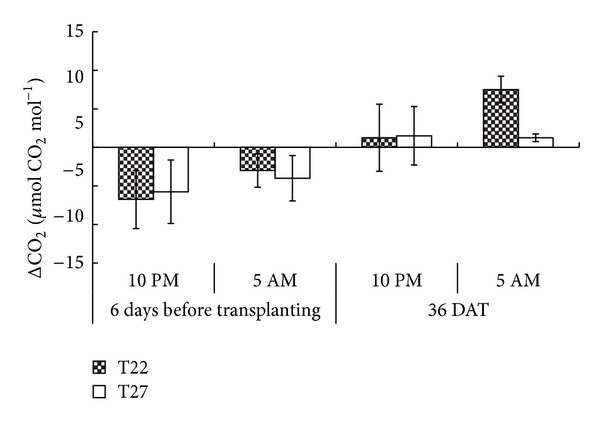
Difference of nighttime CO_2_ concentrations between T22/T27 and ambient conditions before transplanting and at 36 days after transplanting. Error bars are SE. (*n* = 4 replicates).

During daytime, the CO_2_ concentration inside the chamber was similar to that of ambient. To minimize the CO_2_ gap between the chambers and ambient control at night, four exhaust fans were installed to facilitate air exchange inside and outside the chambers. Using the Li-Cor-6400 gas exchange system, CO_2_ concentrations inside and outside the chambers were measured at 10 PM and 5 AM. Changes in CO_2_ concentration between the ambient conditions and controlled-temperature chambers at different time of measurement are shown in Figure 4. A clear deviation in CO_2_ concentration was observed before transplanting in both chambers, which was 5 *μ*mol CO_2 _mol^−1^ lower than ambient CO_2_. At 36 DAT, the change in CO_2_ concentration inside the chambers was negligible at 10 PM; however, CO_2_ concentrations increased at 5 AM the next day (Figure 6). Generally, CO_2_ concentration in the greenhouse or chamber increases at night because of plant respiration. Therefore, the increase in CO_2_ concentration depends largely on the number of plants in the chamber and the rate of respiratory activity of these plants. On the other hand, the CO_2_ concentration at 36 DAT in the ambient is usually higher, that is, 384 *μ*mol CO_2_ mol^−1^ at nightfall, but the difference of 10 *μ*mol CO_2_ mol^−1^ was regarded small enough to alter the effect of temperature on plant.

### 3.5. Plant Response to Elevated Night Time Temperature

In the 2009 dry season, the field chambers were used in screening varieties for tolerance to warm night temperature. Twenty-five varieties from different countries were screened. Among these, varieties sensitive to and tolerant of high night temperature were identified. These varieties are being used for further studies to understand the physiological mechanism behind the genotypic variation and to determine yield response using a bigger plot size. The responses of grain filling rate and yield of IR 72 and IR8 to the field chamber were selected and showed in Table 2. Generally, ambient control produced the relative higher grain filling rate and yield than T22 and T27, while the extent was varied with varieties. The plants responses to elevated nighttime temperature were variable for IR72 and IR8. IR72 was identified as the tolerant variety because little difference of grain filling rate and yield between T22 and T27 were found. Oppositely, IR8 was identified as sensitive one due to its grain filling rate and yield in T27 was significantly lower than that in T22.

#### 3.5.1. Cost Efficiency of the Field Chamber System

The components required for the 16 chambers were listed in Table 3, as well as their cost. It cost about 11800$ in all to setup the system at the beginning, including the body structure, the air-conditioner, and the fans. The body structure and the air-conditioner seized the major parts of the cost. However, once it is setup, the maintainance fee is quit cheap. Recently, infrared heaters (IRH) were reported to be used over open-field plots. However, the amount of energy required in IRH is in response to soil moisture conditions, light intensity, temperature, humidity, and wind speed, and cost of maintainance is significantly greater than the field chamber. As a low-cost field experimental system, our chamber provided a promising alternative to warm the nighttime temperature.

## 4. Conclusions

Nighttime temperatures have been projected to decrease yield more than daytime temperatures. It is necessary to develop climate-ready plants that will adapt to such increases in temperature in order to maintain high productivity under the anticipated climate change. Such studies are best done *in situ*, owing to the limitations of the greenhouse or the phytotrons in terms of area and environmental conditions. In the present system that we developed, rice plants grown under high or low nighttime temperatures were exposed to environmental conditions during the daytime, which were similar to those of the ambient control. Moreover, any unusual alteration in RH and/or VPD or CO_2_ due to changes in canopy air temperature that may affect the plant's tolerance for abiotic stresses (e.g., the dramatic “CO_2_ shock difference”) was minimized during nighttime. This utilitarian system equipped only with night covers, an air conditioner/heater, and several exhausting fans can maintain uniform temperature distribution and relatively constant RH. The system has been used and validated for two seasons in experiments to determine the effect of high night temperature on yield of rice and genotypic variation in sensitivity to high night temperature. The results confirm that low-cost field chambers equipped with air conditioner were effective in studying effects of night temperature on rice crop with minimal perturbation on the other environmental factors.

## Figures and Tables

**Table 1:** Effects of the field chamber system on water temperature. Data are mean ± SE of four replicates.

	Before transplanting (°C)	36 DAT (°C)
Ambient	27.3 ± 0.12b	27.1 ± 0.63a
T22	27.1 ± 0.97b	26.5 ± 0.64a
T27	28.6 ± 0.21a	27.8 ± 0.32a

Mean in column with different small letter was not significantly different between treatments.

**Table 2:** Effects of the field chamber system on rice grain filling rate and yield of IR72 and IR8 in 2009 DS. Data are mean ± SE of four replicates.

Variety	Temperature	Grain filling (%)	Grain yield (g per m^2^)
IR72	T22	74.0 ± 3.22a	443.4 ± 61.07a
	T27	68.8 ± 5.11a	422.1 ± 81.12a
	Ambient	81.7 ± 5.11a	493.3 ± 55.94a
IR8	T22	68.1 ± 6.42a	318.1 ± 70.73a
	T27	37.8 ± 2.69b	106.9 ± 24.61b
	Ambient	77.9 ± 3.36a	426.5 ± 62.55a

Mean in column with different small letter was not significantly different between treatments.

**Table 3:** Components of the chamber and their cost in IRRI, Philippines.

Components	Cost
Greenhouse structure	5000$
UV-transparent film	400$
Construction and electrical supplies	1000$
Exhaust fan	400$
Air conditioner	5000$

Total	11800$
